# COVID-19 mitigation by digital contact tracing and contact prevention (app-based social exposure warnings)

**DOI:** 10.1038/s41598-021-93538-5

**Published:** 2021-07-13

**Authors:** Germán J. Soldano, Juan A. Fraire, Jorge M. Finochietto, Rodrigo Quiroga

**Affiliations:** 1 Instituto de Investigaciones en Fisico-Química de Córdoba (INFIQC-CONICET), Córdoba, Argentina; 2 Instituto de Estudios Avanzados En Ingenieria y Tecnología (IDIT-CONICET), Córdoba, Argentina; 3 Universidad Nacional de Córdoba, Facultad de Ciencias Químicas, Córdoba, Argentina; 4 Universidad Nacional de Córdoba, Facultad de Ciencias Exactas, Físicas y Naturales, Córdoba, Argentina; 5 Saarland University, Saarland Informatics Campus, Saarbrücken, Germany

**Keywords:** Computational models, Viral infection, Health policy

## Abstract

A plethora of measures are being combined in the attempt to reduce SARS-CoV-2 spread. Due to its sustainability, contact tracing is one of the most frequently applied interventions worldwide, albeit with mixed results. We evaluate the performance of digital contact tracing for different infection detection rates and response time delays. We also introduce and analyze a novel strategy we call contact prevention, which emits high exposure warnings to smartphone users according to Bluetooth-based contact counting. We model the effect of both strategies on transmission dynamics in SERIA, an agent-based simulation platform that implements population-dependent statistical distributions. Results show that contact prevention remains effective in scenarios with high diagnostic/response time delays and low infection detection rates, which greatly impair the effect of traditional contact tracing strategies. Contact prevention could play a significant role in pandemic mitigation, especially in developing countries where diagnostic and tracing capabilities are inadequate. Contact prevention could thus sustainably reduce the propagation of respiratory viruses while relying on available technology, respecting data privacy, and most importantly, promoting community-based awareness and social responsibility. Depending on infection detection and app adoption rates, applying a combination of digital contact tracing and contact prevention could reduce pandemic-related mortality by 20–56%.

## Introduction

The COVID-19 pandemic has challenged health authorities around the world since December 2019. Many governments immediately implemented physical distancing and self-isolation measures, ranging from simple “stay-at-home” recommendations to strict lock-downs^1^. Although straightforward, fast and effective in controlling the propagation of the virus, rigorous lock-downs are emergency measures which imply profound economical and social consequences, and cannot be sustained over long periods of time, specially in underdeveloped and developing countries. Sustainable and widely applied nonpharmaceutical interventions such as effectively communicating prevention measures, cancellation of large-scale public gatherings, widespread/mandatory mask utilization, and travel restrictions have proved to be insufficient to contain viral spread in many countries^2^.

In this context, *Contact Tracing* (CT) has been extensively used to attempt to control outbreaks^3^ by identifying and isolating close contacts of diagnosed patients as soon as possible, to prevent further transmission. However, the efficiency of the approach in diminishing COVID-19 propagation strictly depends on how *quickly*, *broadly*, and *accurately* the contact tracing process is^4^. In particular, it is crucial to minimize delays in diagnostics, contact determination and detection, as well as the subsequent isolation of all possibly infected individuals^5^. This argues in favor of so-called *digital* CT, where smartphones automatically store and report contact information^3^ using mainly Bluetooth Low Energy (BLE) technology for proximity detection between devices^6^. The effectiveness of CT has been enhanced by embracing this technology in several countries^7–9^, although not free of data privacy concerns, among other controversies^10,11^.

Both manual and digital CT evidenced a common disadvantage intrinsic to the very nature of this reactive strategy: it depends largely on the percentage of infected individuals which are successfully and quickly diagnosed with COVID-19. However, this issue has been scarcely documented and noted by the community, even though infection detection rates are estimated to be below 12% for most countries, and 16% or less even for developed countries such as the United States of America, Canada, China, Sweden and The United Kingdom^12^. Accordingly, an analysis for the city of New York estimates an Infection Detection Rate (IDR) of 15–20%^13^. Undetected infections are a key characteristic of the COVID-19 pandemic that severely impacts CT strategies, as no contact tracing is possible without diagnosis, which is most generally triggered by symptom onset. We argue this CT limitation is the main reason for observing satisfactory results only when combined with other policies such as detection and isolation via enhanced/random testing or contact avoidance via household quarantine^14^.

In this work, we analyze the impact of different IDRs and time delays on the effectiveness of CT. With the aim of reducing the dependency on these factors, while improving data privacy, we introduce community-based *Contact Prevention* (CP). CP is a novel strategy that attempts to diminish viral transmission by warning users from infection risks due to their current social activity. To quantitatively assess CT and CP in realistic COVID-19 scenarios, a detailed COVID-19 simulation model based on agents, which we named SERIA, is presented. This model leverages several COVID-19 statistical distributions such as social and household contact profiles, IDRs, population age, viral latency period, and fatality rates. We then evaluate and compare the effect of CP and CT strategies on final epidemic size (FES) and mortality (deceased agents as a percentage of the total simulated population).

## Methods

SERIA is a Monte Carlo agent-based model that reproduces the essential aspects of social and household contacts in the context of COVID-19 (SERIA is open-source and publicly available at https://bitbucket.org/juanfraire/seria). The model includes all agents of the well-known SEIR models^15^ and adds an *asymptomatic* agent, a fundamental component of the COVID-19 pandemic^16^. We have used SERIA to simulate a population that has common mitigation measures in place, such as bans on large-scale gatherings, mandatory face masks, and limitations on the size of social gatherings.

To this end, we have parameterized the amount of daily close contacts, as well as the probability of transmission upon their occurrence, so that SERIA averages an effective R of 1.5. Based on this scenario, we evaluate the theoretical performance of CT and CP in terms of FES and mortality rate, according to varying degrees of diagnostic/isolation delays, app adoption and IDRs. Results presented here for each set of parameters correspond to the average of 60 simulations of 1 × 10^5^ agents. Social and household transmission are handled separately. Essential aspects of the model are summarized below; detailed explanations, algorithms and validation analysis are given in the Supplementary Information.

### Agents

**Figure 1:**
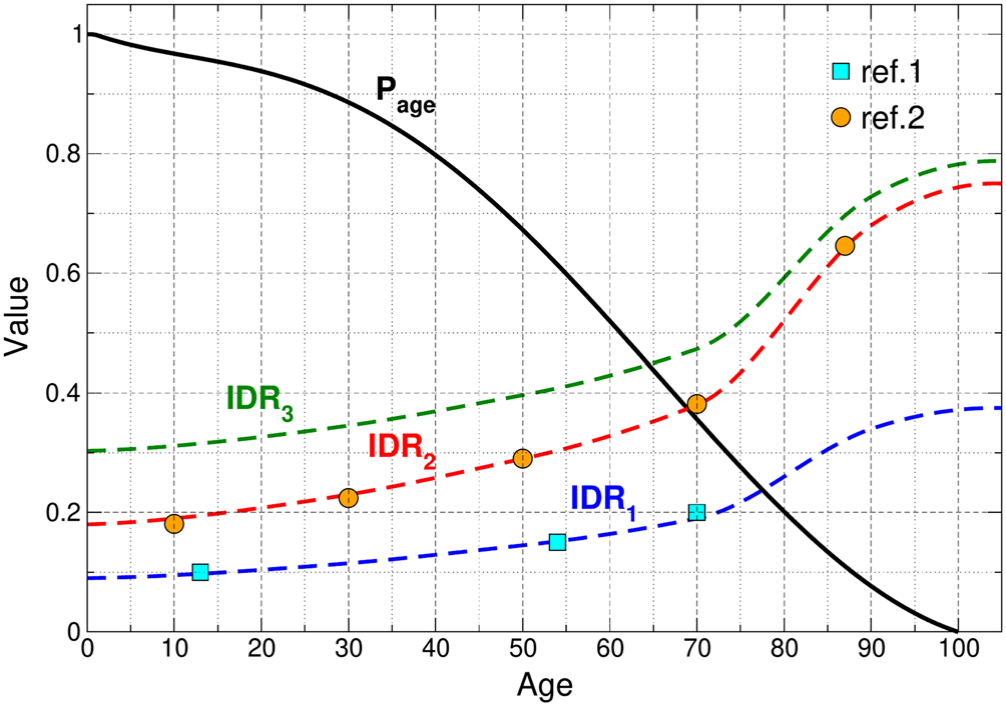
Age distribution function $P_{age}$ and IDR as a function of age for the three scenarios considered in this work: one in which half of symptomatic cases are detected (IDR_1_), one in which all of them are (IDR_2_), and one in which all the symptomatic and 15% of the asymptomatic/oligosymptomatic cases (for each age) are detected (IDR_3_). Ref. 1:^13^ and Ref. 2:^20^.

 We use age as the main defining feature for agents, since it affects all other agent features. For instance, lethality amongst infected, as given by the Infection Fatality Rate (IFR) (see Fig. S4), and social contact patterns S7. Age is assigned randomly to each agent following a probability distribution given by $P_{age}$, shown in Fig. 1. This function was fitted from the latest Argentinian census conducted in 2011, but could be adjusted to any other population age. In SERIA, we divide infected agents into two categories, symptomatic (poly-symptomatic, which present multiple symptoms compatible with COVID-19, and are therefore subsequently tested) and asymptomatic (whether truly asymptomatic or oligosymptomatic, which present none or mild symptoms, and thus are not tested). Symptomatic agents are assumed to self-isolate on symptom onset. We also assume all agents have equal transmission probability upon close contact with a susceptible agent. While some meta-analysis studies have found lower secondary attack rates for asymptomatic subjects^17^, a recent study found similar viral load and infectiousness for PAMS (pre-symptomatic, asymptomatic, and mildly-symptomatic) subjects^18^, and no significant differences have been found in some recent contact tracing studies^19^. Also, the same distribution of symptomatic and asymptomatic infections is used in all simulations. This distribution depends on agent age and coincides with the function that describes IDR2 in Fig. 1 (estimated from Polettis results^20^). As observed in the graph, the probability of symptomatic infection increases with age.

### Time periods

 Once infected, agents sequentially transit the *latency* and *infectious periods*, being able to spread the virus only in the latter. The length of the latency period is assigned randomly to each agent, obeying a log-normal distribution that varies between 1 and 20 days^21^, with a median of 4 days^22^. The infectious period begins when the latency period ends, with a fixed length of 14 days. Transmissibility was assumed to correlate with viral load^23^ and is modeled as a log-normal distribution following experimental determination of viral load kinetics^24^. Transmissibility peaks at day 1.5 post-latency period (before symptom onset)^25^ and then decreases rapidly (see function $f_3$ in Fig. S5). Functions controlling these periods are given in Fig. S5.

### Households

 Household sizes from 1 to 8 are built associating agents so that a given distribution of household sizes and age classes per household is fulfilled. Again, the distribution corresponds to the Argentinian census conducted in 2011. The proportion of homes with 1 to 8 + members are: 18%, 23%, 20%, 18%, 10%, 6%, 2% and 3%, correspondingly. Children, adults and elderly distributions in households are given in Fig. S6.

### Household interactions

 Household interactions are modeled by simulating daily close contacts between all household members.This results in household cohabitants having an infection probability which is 2.4 times higher than for social contacts.

### Social interactions

 Three fundamental aspects emerge from experimentally observed social contact patterns^26^: (i) Young adults have the largest close contact frequency, (ii) Probability of close contact diminishes with increasing age difference between agents (agents are selective, that is, they tend to have close contacts with agents of similar age), (iii) Close contact age heterogeneity increases with age (young agents are more selective than older agents).

These three aspects of social interaction are incorporated into SERIA through the probability function $P_{soc}$, which depends on age and age difference. By means of Monte Carlo simulations, we model social close contacts by randomly drawing two agents and accepting them with a probability $P_{soc}$, according to the ages and age differences of such agents.

### Viral transmission

 If an infected (symptomatic or asymptomatic) and a susceptible agent get in contact, transmission occurs with probability $P_{ctg}$. The latter changes during the infectious period, following a log-normal distribution^27^, peaking at 1.5 days post-latency period, approximately 1 day before symptom onset. $P_{ctg}$ is multiplied by 0.1 in case the infected agent is isolated. This is to account for the fact that perfect self-isolation is not realistic, but also that most violations observed are infrequent and constitute low risk activities^28,29^.

### IDR scenarios

 In order to make a direct comparison between the performance of CT and CP strategies for different IDR scenarios, the same proportion of symptomatic (poly-symptomatic) and asymptomatic (oligosymptomatic and proper asymptomatic) was considered in all the simulations performed (see “Agents” section). IDR impacts directly on CT performance since tracing and isolation are only triggered by detected cases. Because symptomatic individuals are more likely to get tested, and the proportion of symptomatic infections increases with age, the IDR also increases with age. Finally, IDR also relies on testing capacity.

 Although still debated, some studies estimate IDR can be as low as 10% for youngsters and 40% for the elderly even in resource rich cities within highly developed countries as is the case of New York city^13^.

 To analyze scenarios with low, medium, and high detection capability, three IDR curves were considered:

1. IDR_1_: (13% on average) Corresponds to the median IDR estimated for 91 countries^12^. We model this scenario by detecting only half of poly-symptomatic cases. Consequently, only half of poly-symptomatic agents trigger contact tracing, the other half is not tested/detected despite being self-isolated.
2. IDR_2_: (26% on average) This IDR is only reported for 18 out of the 91 countries^12^. In SERIA, corresponds to detecting all symptomatic agents.
3. IDR_3_: (37% on average) Very few countries are estimated to have IDRs above 37% as of June 2020^12^, although testing capacity has greatly improved in most countries since then. We model this scenario by detecting all symptomatic as well as 15% of asymptomatic and oligosymptomatic infections, which are randomly selected.

### Contact tracing

 SERIA comprises manual contact tracing (mCT), which is applied to all detected cases and digital contact tracing (dCT), which only applies to those with an installed smartphone app^30,31^. Household and non-household contacts are traced equally. While the extent of mCT strictly depends on the IDR, dCT also depends on the app adoption rate (*A*). Manual and digital CT have different close contact detection probabilities: For mCT, we assumed that a 40% of the close contacts of a detected case are traced and isolated. For dCT, in order to be detected, both agents in close contact have to have the app installed on their devices. Even in such a case, the wireless beacon (i.e., BLE) emitted by the device might not be successfully detected and interpreted as a close contact by the other end. In this work we consider an accuracy of 85% for detecting close contacts, which corresponds to typical values reported in the literature^30^. Therefore, the actual close contact detection probability for dCT is $A^2 \times 0.85$. Furthermore, our model assumes that testing information of diagnosed cases is made available to the health authority directly by the laboratory (i.e., users are not reporting the test diagnosis via the app). This event then triggers mCT or dCT for every agent diagnosed as positive for SARS-CoV-2. Both mCT and dCT are affected by the time delay *D*, which corresponds to the days from testing to the isolation of a positive case and its contacts. Symptomatic agents are tested and isolated upon symptom onset. In the case of IDR_3_, 15% of oligosymptomatic and asymptomatic agents are detected, isolated and also trigger CT. Close contacts are isolated *D* days after testing of the index case, regardless of the index case category (symptomatic or asymptomatic).

We consider both one-step contact tracing as well as recursive contact tracing (rCT), where not only direct contacts of positive cases are traced, but also contacts of contacts (also known as two-step contact tracing)^32,33^. We assume 100% sensitivity and specificity for PCR testing, and unlimited CT resources/facilities.

### Contact prevention

 CP is possible only using digital means; thus, like dCT, the close contact detection probability is $A^2 \times 0.85$, where *A* is the app adoption rate.

In contrast with CT, where contacts must be able to be *linked to an identity*, CP contacts only need to be *counted*. The average number of daily contacts of each user is then compared with the close contact threshold $C_{max}$ recommended by the authorities through the app. If it is higher, a warning message is sent. In response, agents may reduce their social contact frequency. In doing so, if the average contact count gets below $C_{max}$, the warning expires and the user returns to its normal social habits. The response of the population to the app warning is determined by the non-adherence parameter $d_{a} \in [0,1]$, which moderates the forthcoming contacts after the warning is received. For instance, if $d_{a}=0.6$, after receiving the app warning an agent will decrease its social contact frequency to 60% of its regular rate. $d_a$ is assigned randomly to each agent using a Gaussian distribution.

The resulting impact of CP on the contact frequency of the affected population (which depends on app adoption rate) is shown in the Supporting Information. We can briefly describe the population response to the app warning as follows: Upon app warning 5% of agents decrease their contact frequency to the 0–25% range, 25% of the population decreases contact frequency to the 25–50% range, 45% of the population decreases contact frequency to the 50–75% range, and the remaining 25% of the population decreases their contact frequency to the 75–100% range. This attempts to realistically consider both adherence and capability of agents to reduce their daily close contact frequencies.

The latter was studied for $C_{max}$ from 40 to 90% the maximum average of daily close contacts among age groups (1.5 to 3.5 contacts per day, correspondingly). Each household contact is fractionally counted to avoid members of large households reaching the threshold with few social contacts. In the rCP strategy, the app can also count indirect contacts and warn their users if they reach a recursive close contact threshold $rC_{max}$.

### Figures

Figures were generated using xmGrace version 5.1.22 (https://plasma-gate.weizmann.ac.il/Grace/), and GIMP version 2.10.24 (https://www.gimp.org/).

## Results

To assess the effectiveness of each strategy in diminishing viral propagation, we perform 365 days of SERIA simulation with initial R_*e*_ = 1.5, and assess the percentage of the population infected at the end of said simulations (FES). R_*e*_ = 1.5 is an estimated R_*e*_ for populations that are implementing mandatory mask use, have closed places of worship, schools and universities, banned social gatherings of more than 10 people and implemented protocols for restaurants, bars and gymnasiums. Scenarios analyzed are organized following the aforementioned IDRs distributions, namely 13%, 26% and 37% (overall percentages). For each scenario, the performance of CT and CP strategies are compared for different delays (*D*) and close contact thresholds ($C_{max}$), respectively. The effects of app adoption rates (*A*) are also studied together with the implementation of a combined CT + CP strategy.

### Effect of IDR and delay on CT and rCT

**Figure 2:**
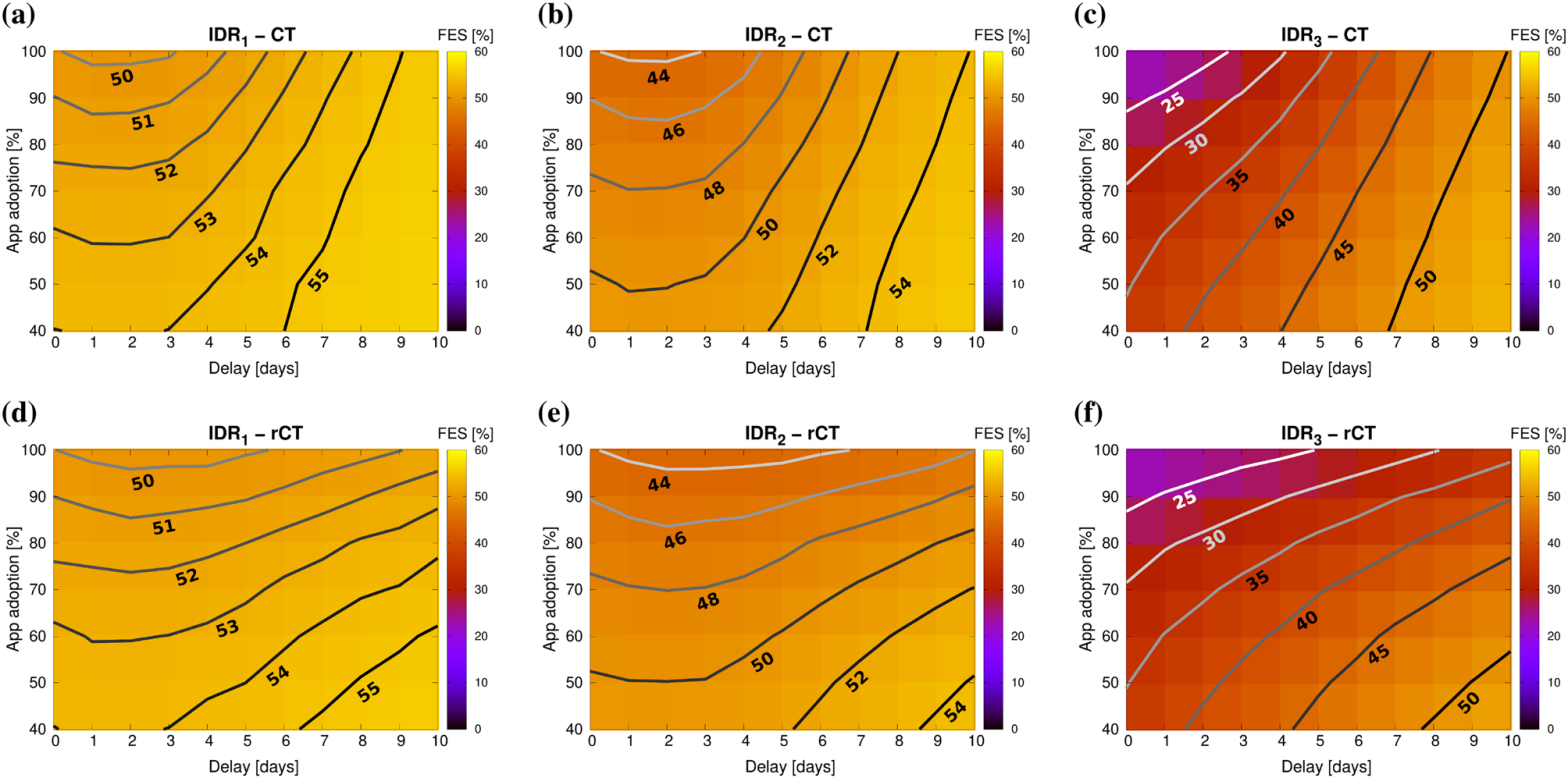
Final epidemic size shown as a heatmap, as a function of CT app adoption and delay (days from symptom onset to contact isolation). The performance of CT (above) and rCT (below) are compared for the three IDR scenarios detailed in Fig. 1. Contour lines for different FES values are shown. Greater delays, low app adoption, and low IDRs result in high FES values, which indicate low CT effectiveness.

Figure 2 presents the impact of app adoption rates and delays on the effectiveness of CT for each of the IDR scenarios.

The most significant result is the strong effect of IDR on CT effectiveness. For the IDR_1_ scenario, CT hardly reduces viral propagation, even for high app adoption rates and low delays. Therefore, adequate diagnostic testing is a requirement for effective CT. CT effectiveness increases for IDR_2_, and most notably, for IDR_3_. These results could explain the limited success observed for digital CT techniques in reducing the spread of COVID-19 in the first half of 2020^34^, when diagnostic capabilities were still low (with IDRs similar to, or even lower than, IDR_1_). The impact of time delays on rCT effectiveness is lower than for CT. At low delays, however, both CT and rCT show practically the same performance for different app adoptions. Thus, rCT may result particularly convenient in cases with high diagnostic/isolation delays.

### CT versus CP and combined strategies

**Figure 3:**
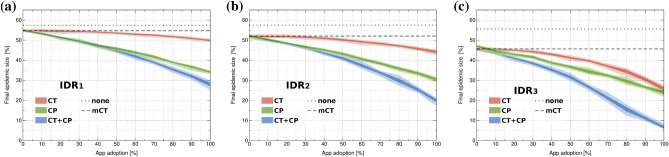
Final epidemic size for CT, CP, and combined CT + CP strategies in three IDR scenarios for varying app adoption percentages. IDR_1_, IDR_2_ and IDR_3_ correspond to 13%, 26% and 37% overall infections detected. We also plot no strategy (none) or only manual CT (mCT) scenarios as dotted lines for reference. Darker and lighter colored areas show the standard deviation and highest/lowest values obtained from 60 simulations of 1 × 10^5^ agents per point. Results correspond to maximum number of direct close contacts $C_{max}=3.1$ for CP and a delay of $D=3$ days since symptoms onset for CT.

Figure 3 shows the performance of CT, CP and CT + CP strategies for IDR_1_, IDR_2_ and IDR_3_ scenarios. To this end, we have fixed parameters to a delay of 3 days in CT and a close contact threshold of $C_{max}=3.1$ in CP. The rather low value of *D* is quite optimistic, while the value of $C_{max}$ corresponds to 80% of the maximum close contact frequency. As expected, the no strategy (none) and mCT FES values are constant for all app adoption rates. However, detecting a higher percentage of infections (higher IDRs) increases the effectiveness of mCT, resulting in lower FES values for IDR_2_ and IDR_3_. At zero app adoption, CP and CT strategies are as efficient as mCT. For IDR_1_, CT is almost insensitive to app adoption rates, achieving similar FES values to mCT. However, CP reduces FES by up to 20% compared with mCT, which suggests that CP is significantly more effective at reducing viral propagation when diagnostic testing is deficient. On the other hand, CT effectiveness increases when detecting all symptomatic agents (IDR_2_) and most notably, when 15% of asymptomatic agents are also identified (IDR_3_). Nevertheless, CP outperforms CT in all scenarios, and the combined CT + CP strategy proves to be remarkably effective reducing FES from 47–55% to 26–42%.

**Figure 4:**
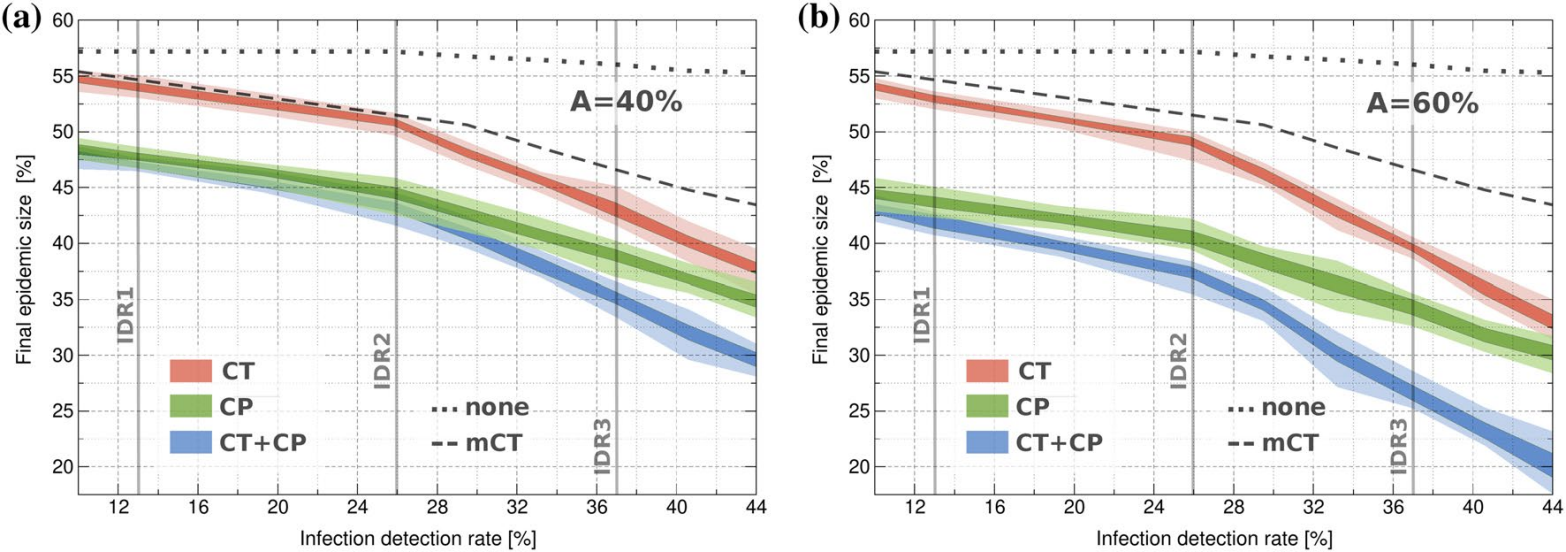
Final epidemic size for CT, CP, and combined CT + CP strategies for varying values of IDR at two fixed app adoption (A) rates. The cases where no strategy (none) or only manual CT (mCT) is applied are plotted as dashed lines for reference (same for both plots). Results correspond to maximum number of direct close contacts $C_{max}=3.1$ for CP and a delay $D=3$ days since symptoms onset for CT. Darker and lighter coloured areas show the standard deviation and highest/lowest simulation values, respectively.

To ensure that these results are not restricted to these particular IDR scenarios, we analyze the effectiveness of CT, CP and CT + CP under varying values of IDR, as shown in Fig. 4. Results confirm that low testing efficiency greatly impairs CT, while CP shows clear improvements with respect to mCT even at low app adoption rates (40%). High IDRs allows mCT to have significant effectiveness in reducing viral spread, although it should be noted we are assuming unlimited mCT resources. As a result, CT + CP achieves a FES reduction of 23% with respect to mCT (for $A=60\%$ and IDR $=44\%$).

Finally, Table 1 summarizes FES, mortality rate, and effective basic reproduction number ($R_e$) for the evaluated IDR scenarios. Without applying any mitigation strategy (none), our simulations estimate a FES of 57% for IDR_1_ and IDR_2_ and 56% for IDR_3_, suggesting that massive testing has little to no effect without contact tracing. Being insensitive to IDR, CP is capable of reducing $R_e$ from 1.52 to 1.36 (for $A=60$% and IDR_1_). Details shown in Fig. S12 reveal that, in contrast with CT, CP lowers $R_e$ from the start. Combined CT + CP strategies can reduce mortality by 28% (IDR_1_), 36% (IDR_2_) and 56% (IDR_3_) with respect to mCT.

**Table 1:** Final epidemic size (FES), mortality rate (M) and effective basic reproductive number (R_*e*_) for three IDR scenarios (average IDR values are given in brackets).

Scenario	A (%)	Strategy	FES [%]	M [%]	R_*e*_
IDR_1_	0	None	57	0.39	1.52
		mCT	55	0.36	1.50
	40	CT	54	0.35	1.49
		CP	48	0.31	1.38
		CT + CP	47	0.31	1.38
	60	CT	53	0.34	1.46
		CP	44	0.29	1.36
		CT + CP	42	0.28	1.31
IDR_2_	0	None	57	0.39	1.52
		mCT	55	0.36	1.50
	40	CT	51	0.33	1.44
		CP	45	0.29	1.36
		CT + CP	43	0.29	1.35
	60	CT	49	0.32	1.42
		CP	40	0.27	1.32
		CT + CP	38	0.25	1.29
IDR_3_	0	None	56	0.37	1.51
		mCT	47	0.30	1.40
	40	CT	43	0.28	1.37
		CP	39	0.25	1.31
		CT + CP	35	0.23	1.28
	60	CT	40	0.25	1.33
		CP	34	0.22	1.27
		CT + CP	26	0.17	1.21

Values for different transmission mitigation strategies and different app adoption rates (A) are compared. FES and mortality rates are calculated at the end of each simulation, while R_*e*_ values are estimated as averages for days 20 to 25 of each simulation.

## Discussion

Although a COVID-19 vaccine is expected to soon be available worldwide as of December 2020 and transmission mitigation will need to persist until herd immunity is achieved, many countries are still seeing increasing amounts of infections. Here we present SERIA, a model which we use to assess CT and CP effectiveness, by contemplating heterogeneous mixing, intricated social interaction patterns and many age-dependant factors such as symptomatic fraction of infections. Our results can offer explanations as to why CT strategies produce conflicting results in different countries, as well as providing compelling evidence that CP is an appealing complimentary approach to control respiratory viruses such as COVID-19.

Our results confirm that long time delays hinder CT effectiveness^5^, but more importantly, they reveal the strong dependency of CT on infection detection rates (IDR), showing very limited effectiveness in low IDR scenarios. This is especially relevant since more than half of countries are estimated to have IDR values similar or inferior to IDR_1_^12^ which renders CT almost completely ineffective. Improving IDR implies increasing diagnostic capacity through infrastructure and sufficiently trained personnel, which requires time as well as large economic investments. For underdeveloped and developing countries, this option may not be plausible.

We developed an alternative mitigation strategy, which circumvents these deficiencies, and could be implemented with low economic requirements, which we named contact prevention (CP). Aimed at promoting community self-awareness, self-control and social responsibility, CP leverages digital assets to inform app users regarding their social contact frequency, warning when social behaviour leads to increased infection/transmission risk.

In contrast to the limitations of CT analyzed above, CP proved to remain effective even for low IDRs, which makes it a particularly interesting strategy for countries with limited testing resources. While FES of CT approaches 55% for low IDRs and 47% for high IDRs, CP achieves FES in the order of 42–26% (at 60% app adoption). Moreover, the combined implementation of CT + CP resulted in ~ 25% FES reduction, bringing mortality down by 28–56% depending on IDR and app adoption rates. CT and CP techniques proved to be rather orthogonal in their contributions to reduce FES. We explain this by the fact that CT excels at quickly isolating detected symptomatic cases and their close contacts, while CP is able to significantly reduce transmission provoked by asymptomatic and pre-symptomatic carriers. Thus, a joint implementation of CT and CP is an appealing approach to mitigate the effects of respiratory virus pandemics.

Moreover, CP presents two further qualitative benefits over CT. One is enhanced privacy of app users, which depending on the CP app configuration (i.e., identify repeated contacts with the same person in rCP) could range from high to full anonymity. The second is long-term game-based habit formation and social conduct modification. The informative notifications from the CP app could provoke profound habit changes that could additionally reduce FES in the long-term, by sustainably making users aware of the risks associated with certain behaviours. Finally, as vaccines are rolled-out, the flexibility of the $C_{max}$ parameter can be conveniently and controllably increased as the population approaches herd immunity.

As on-going work, a prototype for such a CT + CP application is in development by these authors (and others) in the frame of the *ContactAR* project.

## Supplementary information

Supplementary material 1 (pdf 5185 KB)
