# Supplementary material for: COVID-19 mitigation by digital contact tracing and contact prevention (app-based social exposure warnings)
Source: Sci Rep. 2021 Jul 13;11:14421. doi: 10.1038/s41598-021-93538-5 (PMC8277769; doi:10.1038/s41598-021-93538-5)
Supplement: Supplementary file 1 — Supplementary material 1 (pdf 5185 KB) [file 41598_2021_93538_MOESM1_ESM.pdf]

Supplementary Information

Manuscript title: Covid-19 Mitigation by Digital Contact Tracing and Contact Prevention (App-Based Social Exposure Warnings)

Validation

SEIR Model SERIA is capable of reproducing well-known deterministic Susceptible-Exposed-Infected-Removed (SEIR) analytical models[35]. A comparison between SEIR and SERIA simulations show negligible differences (see figure S1). However, most of the features of SERIA (i.e., age-dependency, household grouping, contact tracing, prevention, testing, etc.) had to be disabled for this analysis. In other words, SERIA matches and extends SEIR models in the social and transmission dynamics aspect. As a result, contact tracing logic runs "on top" of a well validated model.

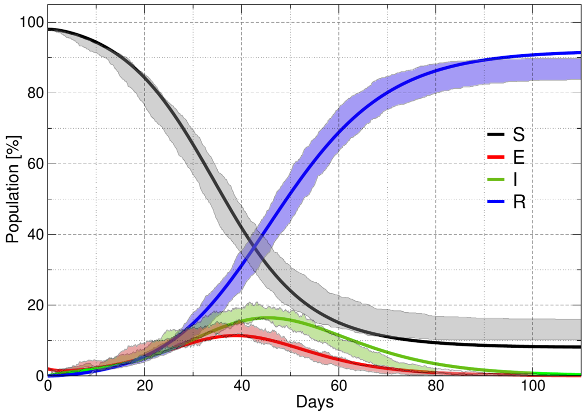

Figure S1. Comparison between a classic SEIR model with a minimalist version of SERIA. ($SERIA_0$). The population percentage of S, E, I, and R agents are display as lines for SEIR, and as areas for $SERIA_0$ (lowest and highest values of 20 simulations of one thousand agents each). A Fortran code was written for the former, in which the SEIR differential equations were solved using the Runge-Kutta method. Parameters for SEIR are: $\mu = 0.; \alpha = 0.2; \beta = 0.34; \gamma = 0.125$ which result in an $R_0 = 2.7$. Parameters for SERIA are: latency period $= 1/\alpha = 5.0$ days; Infection; period $= 1/\gamma = 8.0$ days; transmission probability $= 0.06$.

Age Distribution of Index Cases Another fundamental aspect to validate the accurate reproduction of COVID-19 pandemic is the age distribution of index cases (ADIC). An index case is considered to be the first infected individual within a household. Specifically, ADIC attempts to analyze the age dependency of social viral transmission. In order to evaluate if SERIA is capable of modeling such patterns, simulations with and without a restriction on social contacts (applying the aforementioned social interaction algorithm) were compared.

If no restrictions are applied (all contacts between agents are accepted), the ADIC metric follows a rather smooth distribution, as shown in figure S2a. A gradient of cases is observed from younger to older agents, only due to their larger quantity in the simulation. A completely different pattern is obtained if the probability of contacts is modulated through the probability P_{soc}, as shown in figure S2b. In this case, three distinctive diagonal lines can be observed. The central diagonal corresponds to similar-age contacts such as spouses, siblings, work and social contacts. The top diagonal corresponds mainly to parents infecting children, while the bottom diagonal corresponds mainly to descendants infecting parents. Moreover, diagonals are not symmetric. Instead, more cases are accumulated in the top diagonal, pointing towards a greater chance of adults infecting children than the other way around. Naturally, this trend could be reversed if schools were open. Overall, this transmission pattern obtained from SERIA is in close agreement with reported metrics from two states of India, involving more than half a million individuals[36]. It also resembles estimated spreading patterns of viruses by social contacts[26].

In conclusion, the regulation of contacts through the probability P_{soc} allows SERIA to succeed in mimicking the essential age-dependant aspects of social transmission during the COVID-19 pandemic, which adds to the SEIR model validation presented before.

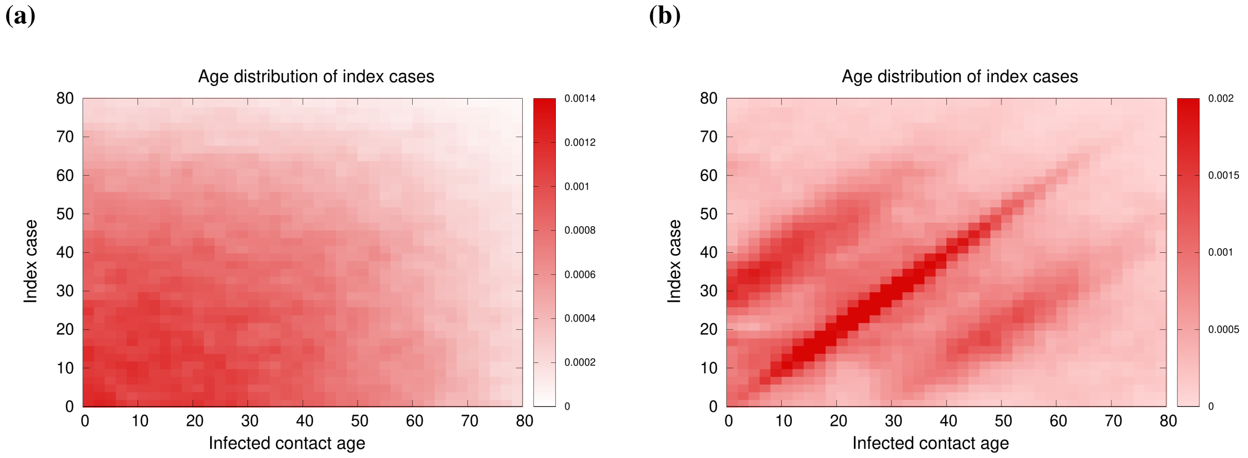

Figure S2. **(a)** Age distribution of index cases (ADIC) obtained if all collisions are considered as close contacts. A rather smooth distribution of transmission is observed. **(b)** ADIC obtained if a restriction (ruled by the probability P_{soc}) is imposed on collisions to be considered as effective contacts. Three diagonal lines emerged, corresponding to similar-age transmission (central diagonal), parents-to-children transmission (top diagonal) and descendants-to-parents transmission (bottom diagonal).

Methods

Agents

There are six agent states in SERIA: Susceptible **S** (have not been infected), exposed **E** (infected, but transits the *latency period* during which they can not transmit the virus), symptomatic **I** and asymptomatic **A** (both can spread the virus during the *infectious period*), recovered **R** (immune after surviving the disease) and deceased **D**. Transitions between states are triggered by agent interaction, as detailed in figure S3.

Age This is the most important agent feature since all others are directly or indirectly dependent of it. Age is assigned randomly to each agent following a probability given by P_{age}, as follows:

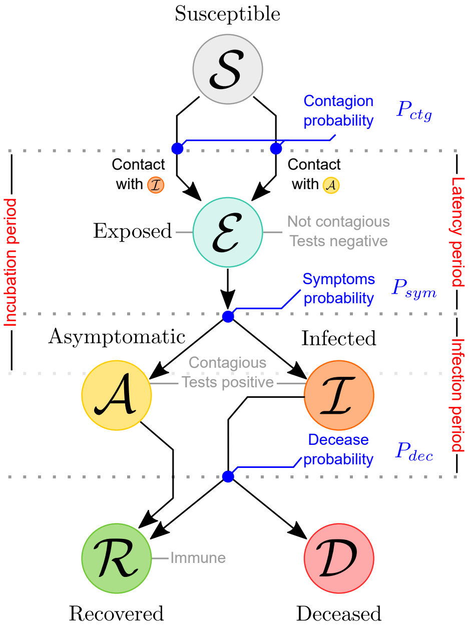

Figure S3. SERIA agents interaction and state transitions.

i) An age x from 0 to 105 is randomly drawn.

ii) Another random number $r \in [0;1]$ is generated.

iii) If $r < P_{age}(x)$, the value of x is accepted, otherwise the procedure is repeated until the condition is fulfilled.

Figure 1 shows the function P_{age} used in this work, which was fitted from the latest Argentinian census conducted in 2011.

State transitions A diagram of agents interactions and state transitions is given in figure S3. Transitions occur under the following circumstances: If **I** or **A** contact **S**, then **S** can become **E**. Then, after an latency period t_{inc}, **E** can either become **A** or **I** following a symptomatic proportion given by IDR_2 (see main article). **A** spreads the virus during the entire infectious period, while **I** does it only up to symptoms onset, after which it is self-isolated. After the infectious period, **A** always evolve to **R**, while **I** agents can either recover, or die as a consequence of the disease (**D**). The latter is governed by the Infection Fatality Rate (IFR) $\in [0,1]$, displayed in figure S4. The IFR used in this work was fitted from Argentinean metrics. In practice:

i) Right after the infectious period of agent i of age x, a random number $r \in [0;1]$ is generated.

ii) If $r < IFR(x)$, agent i dies, otherwise, it recovers and gains immunity.

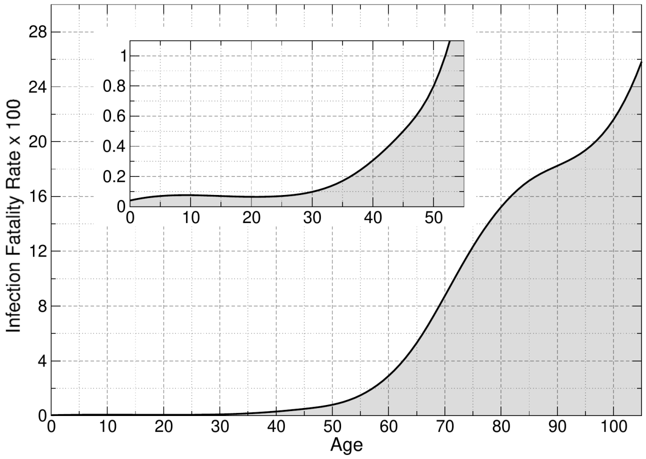

Figure S4. Infection Fatality Rate (IFR) as a function of age. Values are multiplied by 100 to ease the reading of IFR values for ages below 50 years. The inset shows values for agents younger than 55 years old.

Time Periods The time window during which an infected agent is not contagious, is referred to as the *latency period*. The *infectious period* is the time window in which the agent can spread the virus. The *incubation period* is defined as the time window from infection to symptom onset. In some cases, as it is the case of SARS-CoV-2, the infectious period starts *before* the incubation period ends. This means pre-symptomatic transmission is highly probable. Incubation, latency, and infectious periods are sketched in figure S3. The *latency period* t_{lat} varies in-between 2 and 20 days[21], with a peak at around 4 days[22]. A random period with a log-normal probability distribution given by f_{lat} (see figure S5) was assigned to each agent, with the same procedure as the one used to assign ages. A constant length of 14 days was assumed for the *infectious period* t_{inf}, with contagiousness following a similar distribution to what has been determined for viral load, peaking at day 1.5 (before symptom onset) and then decreasing rapidly (see function f_3 in figure S5).

Household simulation Households from 2 to 8 members are built associating agents so that a given distribution of household sizes and age classes per household is fulfilled. In SERIA, agents corresponding to the same household are associated, and are forced to meet each other once a day. In this case, the household distribution corresponds to the Argentinian census conducted in 2011. The proportion of homes with 1 to 8+ household members are: 18%, 23%, 20%, 18%, 10%, 6%, 2% and 3%, correspondingly. Age classes distributions (children, adults, elderly) are given in figure S6.

Interactions

Social interactions Functions f_1 and f_2 control the social contact probability function. The former indicates the relative frequency of contact by age, and the latter indicates the relative probability of contact by age difference (which also changes

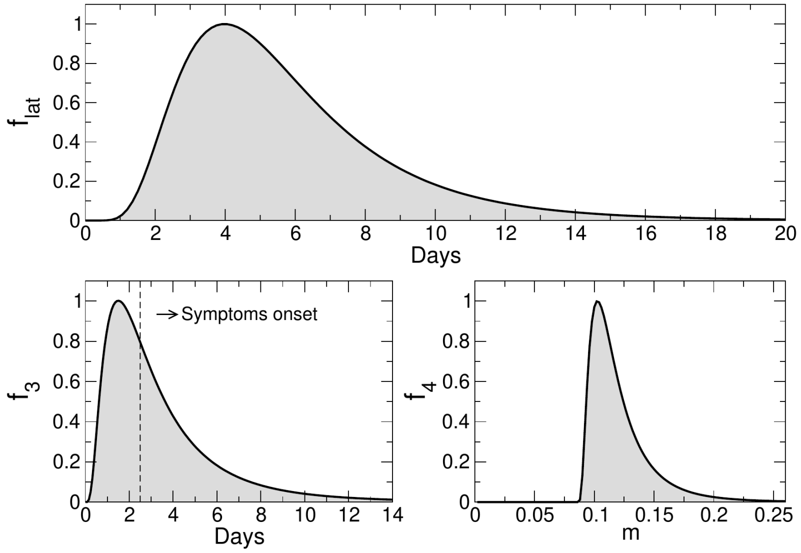

Figure S5. Top: Latency period distribution f_{lat}. A random latency period length is assigned to each agent following such distribution. **Bottom:** Infectiousness profile over time, since the end of the latency period (left). Function f_3 indicates the relative probability of transmission for a certain day with respect to the maximum value (reached at 1.5 days post-latency period, while the median time for symptom onset is 2.5 days post-latency period). The actual probability is given by the product mf_3, where m is the maximum probability of transmission. A random value of m with a probability distribution given by f_4 (right) is assigned to each agent to account for heterogeneity in contagiousness amongst infected individuals.

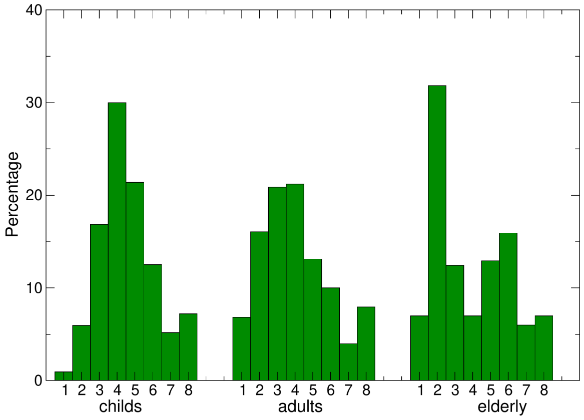

Figure S6. Age class distribution in household sizes from 1 to 8 members. The sum of all the bars for each group gives 100%. Therefore, this graph can also be interpreted as the probability of finding children, adults, and elderly in households from 1 to 8 members. Children, adult, and elderly correspond to agents of up to 20, from 21 to 65, and older than 65 years old, respectively.

with age) such that

$$P_{\text{soc}}(x_i,x_j) = f_1(x_i)\, f_1(x_j)\, f_2(x_{ij},\Delta x) \quad (1)$$

$$\Delta x = |x_j - x_i| \quad (2)$$

where x_i and x_j are the ages of agents in possible contact, x_{ij} their age average, and Δx their absolute age difference. Using the above equations the following algorithm is applied:

i) Two agents i and j are drawn and the corresponding $P_{soc}(x_i,x_j)$ is calculated.

ii) A random number $r \in [0,1]$ is generated.

iii) If $r < P_{soc}$, the i-j contact is accepted, otherwise is rejected.

This procedure is repeated until a given number of contacts N_c is reached. N_c is also used as a metric of time. In a scenario with 1×10^5 agents and 3.0 average contacts per day per agent, N_c is the equivalent of the number of contacts in an hour for that scenario. Since the number of contacts needed for N agents to have an average of 1 contact per agent is $N/2$, we get:

$$N_c = \frac{3.0 \times 1 \times 10^5}{2 \times 24} \tag{3}$$

giving a value of $N_c = 6250$ contacts per hour.

The fitting of functions f_1 and f_2 is shown in figure S7. Using the contact matrices of Mossong's study[26], we processed the data to obtain the frequency of contacts per age relative to the maximum frequency per age. This was done both for ages and for age differences. The fitting of these two resulted in f_1^* and f_2^*, respectively. They correspond to a scenario with opened schools. Since we focus in a scenario with schools closed, we modified them by reducing values for f_1^* and increasing them for f_2^*, in both cases only for ages below 20 years. This results in functions f_1 and f_2, shown as dashed lines in figure S7.

(a)

(b)

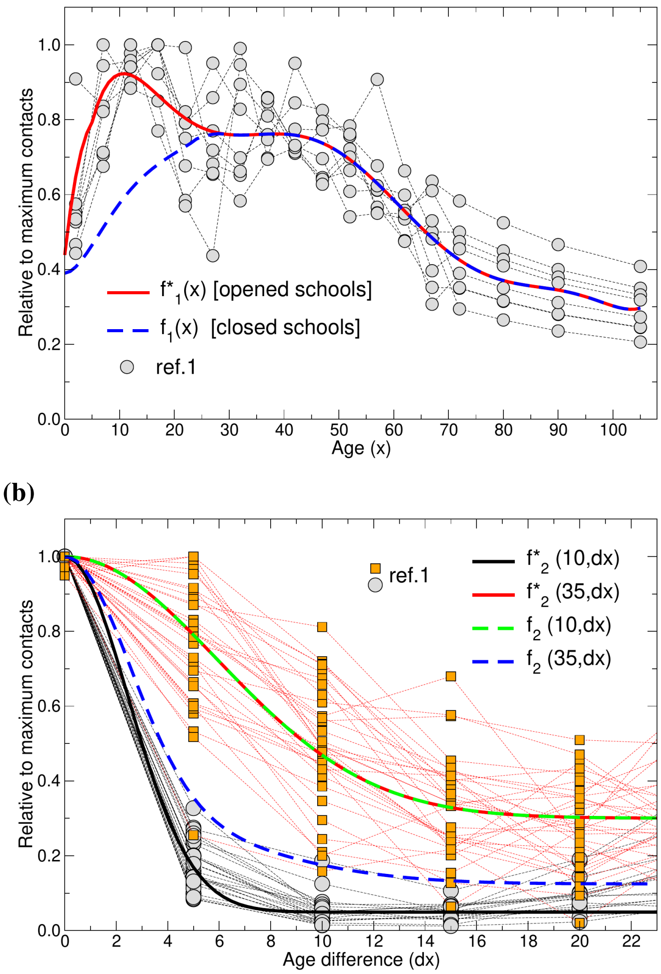

Figure S7. Fitting of functions f_1^* **(a)** and f_2^* **(b)** using ref.1[26]. These functions are later processed to represent the cases of schools closed, resulting in functions f_1 and f_2. The former indicates the relative frequency of contact by age, and the latter indicates the relative probability of contact by age difference (which also changes with age). f_1 and f_2 give shape to the probability of social contact P_{soc}, employed to model social contact patterns.

Total interactions After including social and household interactions, agents can have up to 11 close contacts per day, as shown in figure S8.

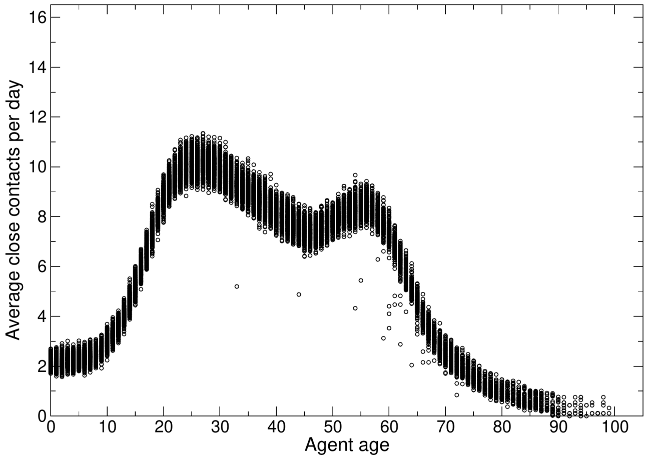

Figure S8. Average close contacts per day (corresponding to the last 7 days) as a function of agent age in a scenario with no CT or CP (no restriction is imposed on the close contact frequency) for a single simulation with 1×10^5 agents.

Viral transmission

If an infected (**I** or **A**) and a susceptible agent get in contact, transmission can occur. The probability that agent i infects agent j is given by $P_{ctg}(i)$ and is assumed to be proportional to the infectiousness of i. The transmission probability $P_{ctg} \in [0;1]$ is controlled by functions f_3 and parameters m and Q by:

$$P_{ctg}(i,d) = m(i)\, f_3(d)\, Q(i) \quad (4)$$

$$f_3(d) = \frac{2.821}{d} \exp\left(-\frac{[\log(d) - 0.97]^2}{1.125}\right) \quad (5)$$

$$f_4(m) = \exp\left(-\left[\ln\left(60(m - 0.08)\right)\right]^2\right) \quad (6)$$

where d are days. While f_3 determines the profile of transmission probability over time, $m \in [0;1]$ determines its scale. Therefore, if $m = 0.1$, the transmission probability P_{ctg} will have the profile of f_3 with a maximum value of 0.1 (10% chance). The parameter m is assigned randomly to each agent with a log-normal distribution given by f_4 using the same procedure as the one used for age assignment. Functions f_3 and f_4 are shown in figure S5. Function f_3 was fitted using an estimation of the infectiousness profile of SARS-CoV-2[27]. Function f_4 was constructed following the observation that a few individuals presented much larger viral load than the average, and it was calibrated to give a reproductive number $R_e = 1.5$.

The parameter Q distinguishes between isolated and not isolated agents, being $Q = 0.1$ in the former case and $Q = 1$ in the latter. In this way, isolated agents have a 10% chance to infect or get infected with respect to non-isolated agents. In comparison with scenarios in which isolation is perfectly hermetic, this is a more realistic approach. In SERIA, symptomatic agents are self-isolated after symptoms onset, while asymptomatic agents are isolated only if tested and diagnosed positive (which is how 15% of asymptomatic are detected in scenario IDR_3 and only there).

In summary, the probability of transmission $P_{ctg}(i)$ depends on how contagious is the agent i (controlled by m), in which stage of the infectious period it is in (controlled by function f_3), and if it is isolated or not (given by parameter Q).

IDR Scenarios

Three IDR scenarios were considered. They all share the same proportion of symptomatic agents, as obtained from Poletti´s results[20]. The only difference between them is whether the actual detected cases are below, the same, or above that proportion. In the former case not all symptomatic cases are reported; in the latter all symptomatic plus some asymptomatic cases (by massive testing) are detected.

1. IDR_1: (13% on average) Corresponds to the median IDR estimated for 91 countries[12]. We model this scenario by detecting only half of the symptomatic cases.

2. IDR_2: (26% on average) This IDR is only reported for 18 out of the 91 countries[12]. In SERIA, corresponds to detecting all symptomatic agents.

3. IDR_3: (37% on average) Only Australia, Russia, Puerto Rico, Thailand, South Korea and Israel are estimated to have IDRs above 37%[12]. We model this scenario by detecting all symptomatic and 15% of the asymptomatic (assumes massive testing).

As stated above, only detected cases can trigger CT. The following algorithm explains how agents are selected to do so:

- IDR_1: From 0 to 10 days after symptoms onset of agent i (depending on the diagnostic/isolation delay) a random number $r \in [0;1]$ is drawn. If $r < 0.5$, symptomatic agent i is detected and CT can proceed from it.
- IDR_2: CT is enabled from all symptomatic agents with a given delay.
- IDR_3: Idem IDR_2. On top of this, a random number $r \in [0;1]$ is drawn for asymptomatic cases. If $r < 0.15$ such agents are detected.

Symptomatic agents The percentage of symptomatic infections by age (x) each age is given by the probability $P_{sym}(x)$ which is exactly the same as function $IDR_2(x)$, shown in figure 1. This function, fitted from Poletti's results[20], is used as follows:

i) At the beginning of the simulation a random number $r \in [0;1]$ is generated for each agent.

ii) If $r < P_{sym}(x)$, where x is the agent age, such agents will develop symptoms if they get infected, otherwise, they will be asymptomatic.

Contact Tracing

The close contact detection probability for mCT and dCT is 40% and 85%, respectively. In practise this implies that:

i) For each contact of an index case, a random number $r \in [0;1]$ is generated.

ii) If $r < 0.4$ (for mCT), or if $r < 0.85$ (for dCT), such a contact will be detected and isolated, otherwise it will not.

The same procedure is applied to contacts of index cases in recursive CT.

Digital CT depends on the parameter A, which is the percentage of the population that downloaded the CT app. To implement this:

i) At the beginning of the simulation a random number $r \in [0;100]$ is generated for each agent.

ii) Agents in which $r < A$ download the app, the rest do not.

iii) Only contacts between agents that have the app are considered. The chances of actually detecting them is 85%, as explained above.

Contact Prevention

The app adoption parameter A is also used for CP. Only contacts with the app are counted and with an effectiveness of 85%. The important difference with CT is that contacts are only counted (the identities of the agents in contact are not stored). Nevertheless, CP apps might profit from identifying household members, which are counted differently in the application. The average number of daily contacts (C) of each user is compared with the close contact threshold (C_{max}) recommended by the authorities through the app. If $C > C_{max}$ a warning message is sent. In principle, C could be obtained by adding social and household contacts for each agent, but this will result unfair for large households since their members will reach the contact threshold even without social contacts. To solve this we define C as:

$$C(i) = C_{soc}(i) + C_{fml}(i) \quad (7)$$

$$C_{fml}(i) = F(n)\, C_{max}(i) \quad (8)$$

$$F(n) = 0.03\, n(i) \quad (9)$$

where C_{soc} and C_{fml} are the average number of daily social contacts and daily household contacts, respectively; $n(i)$ the number of cohabitants of agent i, and F a factor. Equation 8 indicates that household contacts are considered always as a fraction of the the contact threshold. This fraction is given by F which increases with the household size (through n). Table S1 shows all possible values of F in this study. One way of interpreting the above equations is that the more people within the household, the smaller the number of social contacts needed to reach the contact threshold. For example, agents with 4 cohabitants (in 5-member household) will reach the threshold at 88% the social contacts required for agents without cohabitants.

n	0	1	2	3	4	5	6	7
$F(n)$	0	0.03	0.06	0.09	0.12	0.15	0.18	0.21

Table S1. Factor F as a function of the number of cohabitants n for all household sizes considered in this work.

App warning response Agents may reduce their contact frequency, or not, according to the non-adherence parameter $d_a \in [0,1]$, which moderates the forthcoming contacts after the warning is received (i.e., $d_a = 0.6$ implies a 60% of the regular contact rate). Once the average close contact count gets below C_{max}, the warning expires and the user returns to its social habits. This is implemented in SERIA in the following way

i) After a contact between i and j has been accepted as explained above, the probability $W(i,j) = w(i)\,w(j)$ is calculated, where $w(i) = d_a(i)$ if the agent has received the app warning, and $w(i) = 1$ otherwise. Therefore $W(i,j)$ is the probability that i and j *actually* meet after receiving the app warning.

ii) A random number $r \in [0;1]$ is generated.

iii) If $r < W(i,j)$ that contact actually takes place, otherwise it does not. Still, this avoided contact is counted only for purposes of time tracking (it adds to N_c as explained above).

Parameter d_a is assigned randomly following a Gaussian distribution P_d given by

$$P_d = \exp\left(-12.(d_a - d_0)^2\right) \tag{10}$$

where $d_0 \in [0,1]$ determines the distribution of response to the App warning in the population. The higher d_0, the lower the adherence. A few examples of response distributions P_d are shown in figure S9a. In this work we choose to $d_0 = 0.6$. As shown in figure S9b, the corresponding distribution implies that among all the users receiving the warning, only 30% will get in contact with less than half their habitual number of contacts, while 70% will get in contact with more than half that number.

Direct contact threshold CP is affected by the close contact threshold suggested by the authorities (C_{max}). The actual threshold suggested by the app is C_1 defined as

$$C_1 = 0.85\,A\,C_{max} \tag{11}$$

where A is the app adoption threshold. If all the population had the app and detection effectiveness was 100%, C_1 and C_{max} would coincide. In a simulation with 3.0 average daily contacts per agent, the maximum number of daily contacts is 3.9. To analyze a wide variety of cases, we study the performance of CP for C_{max} from 40% to 90% that value (1.5 to 3.5 contacts). As an example, figure S10 shows how the number of direct contacts change in the population by applying the CP strategy. Results correspond to the direct close contact threshold $C_{max} = 3.1$, which is 80% the frequency contact of the most social agents. A substantial reduction of contacts is observed. Nonetheless, a few cases are observed above the direct contact threshold. These cases correspond to agents which only slightly alter their social habits after the app warning, either due to non-compliance, or due to non-work contacts representing only a small fraction of their daily total contacts. In CP no limit is established for indirect contacts (contacts of contacts). Still, because the number of direct contacts was reduced, so did the indirect contacts (see figure S10 top panel).

Recursive Contact Prevention In addition, the CP app can also count indirect contacts (contacts of contacts) and warn their users if they reach an indirect close contact threshold rC_{max}. This is the so-called recursive contact prevention strategy (rCP). In contrast with CP, where household member contact count is modulated, in rCP, all social and household contacts of a direct contact are counted. If the sum of indirect contacts surpass a given recommended value for recursive contacts rC_{max}, another warning is send to app users to which they respond with the same adherence probability explained above. Figure S10 shows how the number of indirect contacts change when applying rCP. Taking the CP strategy as a reference, we observed that rCP reduces further the number of contacts. In this work, we chose rC_{max} to be close and below the maximum value of indirect contacts corresponding to applying CP, as shown in the above example. In general, the rCP approach plays the role of a second barrier to those agents that did not obey the warning.

Results

CP vs CT

Figure S11 shows the FES with varying app adoption A and direct close contact thresholds C_{max} for CP and rCP strategies. IDR_1, IDR_2 and IDR_3 are compared. To ease the comparison we also show the corresponding results applying CT and rCT. By

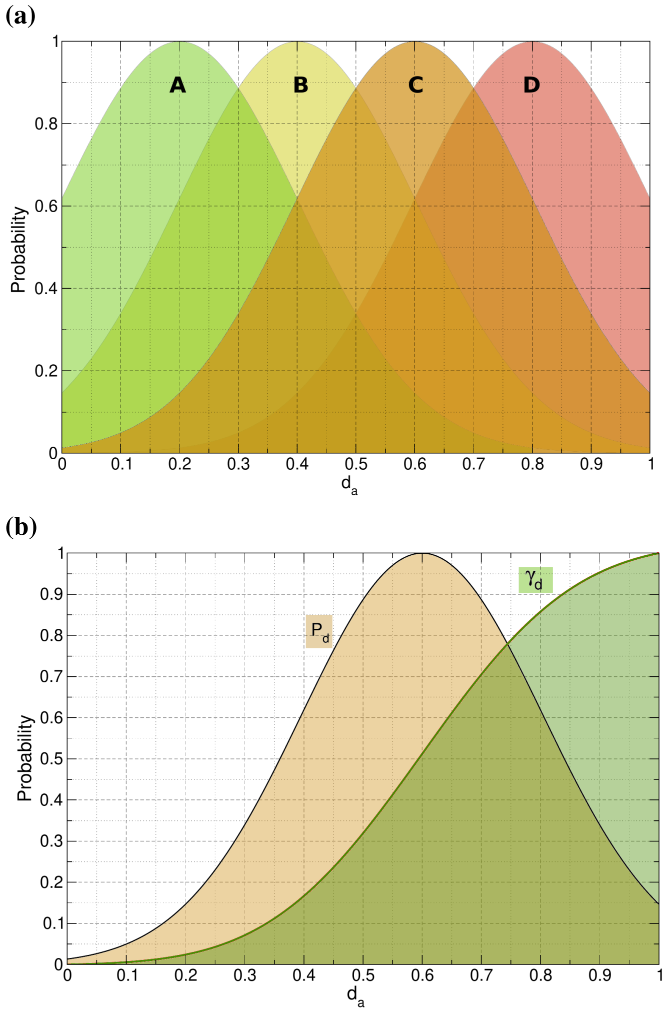

Figure S9. **(a)** Different response distribution functions (A-D). A random value of non-adherence d_a is assigned to each agent following one of these distributions. In this work we used distribution C (P_d). **(b)** Response distribution used in this work (P_d) and its integral (γ_d) scaled so that $\gamma_d(1) = 1$. This integral gives the percentage of the population with a non-adherence parameter up to d_a. For instance, the graph point (0.5;0.3) indicates that 30% of the population has a non-adherence parameter equal to 0.5 or less. This indicates that 30% of users receiving the app warning will reduce their contacts to less than half their usual number of social contacts; the other 70% will contact more than half their usual social contacts.

comparing FES for the same app adoption we observe that CP outperforms CT in all scenarios tested here. In fact, CP at low IDRs outperforms CT at high IDRs. The improvement of rCP respect to CP is subtle and manifests more clearly at low A and high C_{max}.

(a)

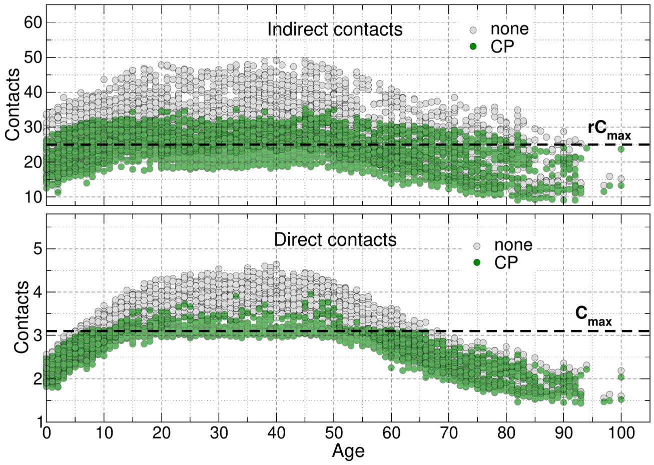

(b)

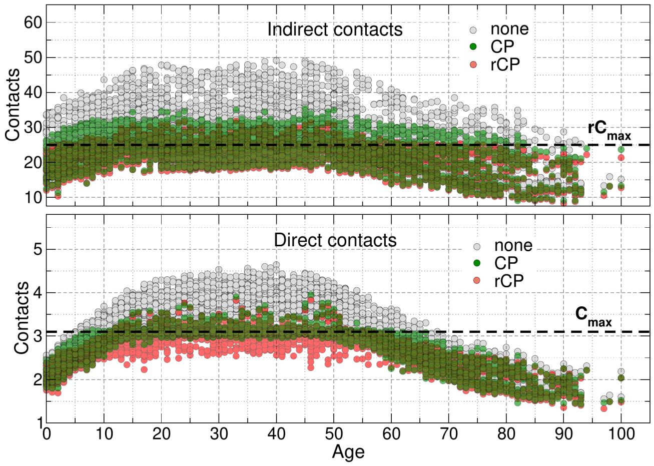

Figure S10. Average of total (social and household) daily contacts as a function of age for each agent. For simplicity, only 4000 points are shown. Direct and indirect contacts (contacts of contacts) are shown separately. Direct and indirect close contact thresholds (C_{max} and rC_{max}, respectively) are shown as horizontal lines. **(a)** Comparison between not applying any mitigation strategy (none) and applying contact prevention (CP). **(b)** Comparison between the last two strategies (non and CP) and recursive contact prevention (rCP).

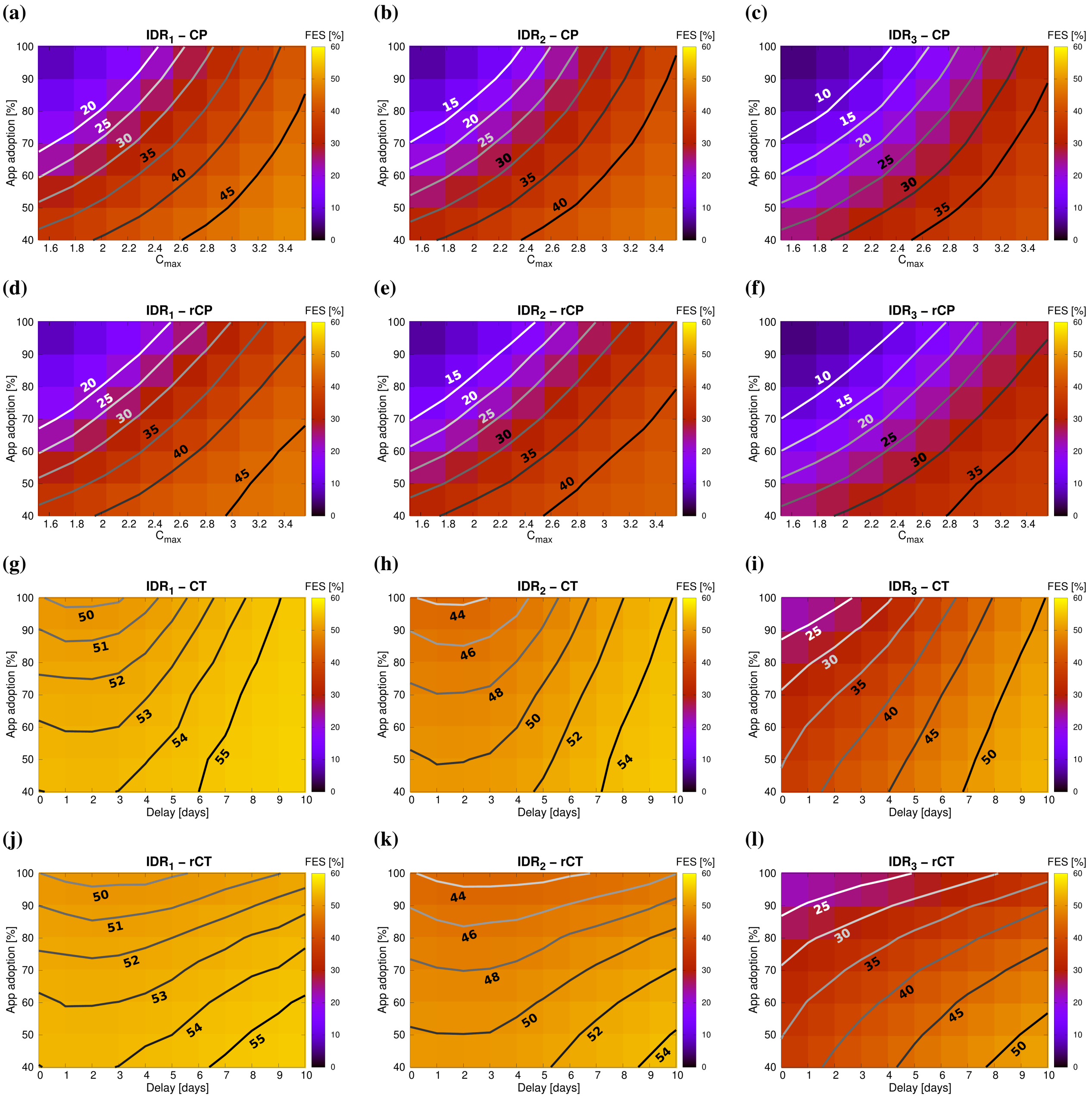

Figure S11. Final epidemic size (FES) of the three IDR scenarios detailed in figure 1 under CP and CT strategies. Contour lines for different FES values are shown. These results correspond to 60 simulations per point (8400 simulations for the 12 graphs). **(a-f)** CP and rCP performance with varying app adoption and direct close contact thresholds C_{max}. **(g-l)** CT and rCT performance with varying app adoption and delay D.

Basic reproductive number

Figure S12 compares the R_e in scenarios IDR_1, IDR_2 and IDR_3 for all the mitigation strategies studied here, with an app adoption of 60%. In comparison with no strategy (none), CT fails to lower R_e in appreciable terms. Although a noticeable drop is observed at day 20, it is still insufficient to significantly reduce R_e. In contrast, CP enables a reduced R_e right from the first days, starting with lower values (1.4 compared to 1.6). CP also shows a drop of similar magnitude than CT at day 20. Similar trends are observed for the combined CT+CP strategy.

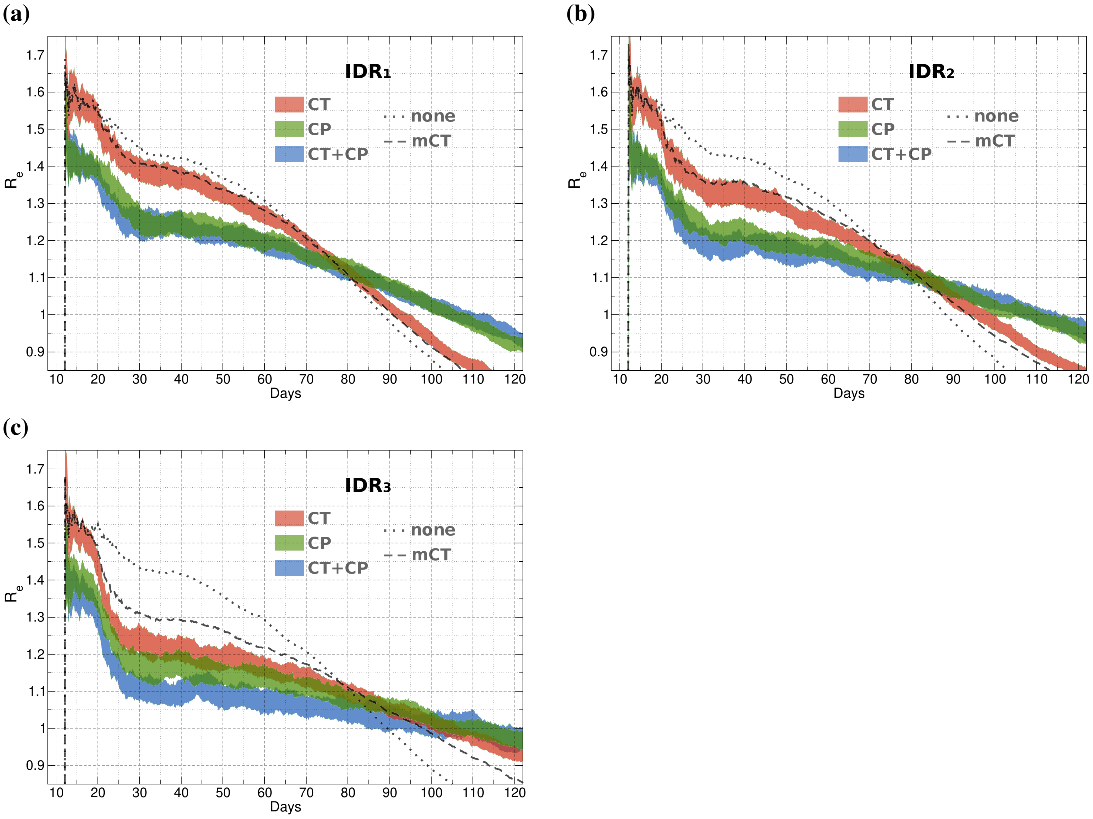

Figure S12. Basic reproductive number (R_e) during the first 120 days of the simulated pandemic. CT, CP, and combined CT+CP mitigation strategies are compared. The case of not applying any strategy (none) and only mCT are also shown as a reference. Results correspond scenarios IDR_1 (a), IDR_2 (b) and IDR_3 (c) at 60% of app adoption with a delay of 3 days (for CT) and a direct contact threshold of 3.1 (for CP).
